# Threatened fertility and gonadal function after a polytraumatic, life-threatening injury

**DOI:** 10.4103/0974-2700.62110

**Published:** 2010

**Authors:** Michael A Ward, Pamela L Burgess, Daniel H Williams, Casey E Herrforth, Michael L Bentz, Lee D Faucher

**Affiliations:** Department of Surgery, University of Wisconsin School of Medicine and Public Health, Madison, WI - 53705, USA; 1 Department of Urology, University of Wisconsin School of Medicine and Public Health, Madison, WI - 53705, USA

**Keywords:** Polytrauma, genital, trauma, fertility, testicle, revascularization, reproduction

## Abstract

Trauma literature regarding management of genitalia trauma affecting future fertility and gonadal function in the face of coexisting life-threatening injuries is underdeveloped. We present a unique case that necessitated integrative management of a 24-year-old male who became entangled within the blades of a manure spreader and presented with life-threatening trauma in addition to severe genital trauma, including penile degloving, bilateral testicular avulsion and bilateral spermatic cord laceration. During the initial stabilization and surgical management, urology and plastic surgery were consulted to assess the urogenital injuries. Together, the surgical team orchestrated potentially life-saving interventions while successfully performing both a testicular sperm extraction and a testicular revascularization. Viable sperm was collected on the day of surgery and initial follow-up showed preserved sexual function and adequate perfusion to the testicle. This report presents a case and provides a review discussing the management of traumatic genital injuries and the importance of early involvement of surgical specialties in genitalia trauma to optimize future fertility and gonadal function. The literature search was performed in August 2008 using Medline for articles only in English, including any of the following terms: polytrauma, trauma, penis, testicle, degloving, avulsion, spermatic cord, laceration, fertility, reproduction or revascularization.

## INTRODUCTION

A level-1 trauma center is required to possess a full range of specialists and equipment to offer the highest level of surgical care to trauma patients.[1] These resources are arranged to orchestrate the resuscitation, surgical intervention and stabilization of the critically injured patient in an organized and efficient manner. T he success of these life-saving interventions typical of a level-1 trauma center methodology is well established.[2] Recently, there has been more focus on the implications of the patient's quality of life post intervention. This report specifically discusses the management of severe genital trauma in a polytraumatic injury.

## CASE REPORT

A 24-year-old man was working on a farm loading bails of manure into the manure spreader when a coworker started the machine for “just a second” to test the motor. The patient became entangled in the spreader mechanism, which created the injury. Local emergency medical services were immediately dispatched. Our patient was treated with established protocols and air ambulance transport was requested due to the severity of the injury. He was transported to our level-1 trauma center in a stable condition.

**Figure 1:**
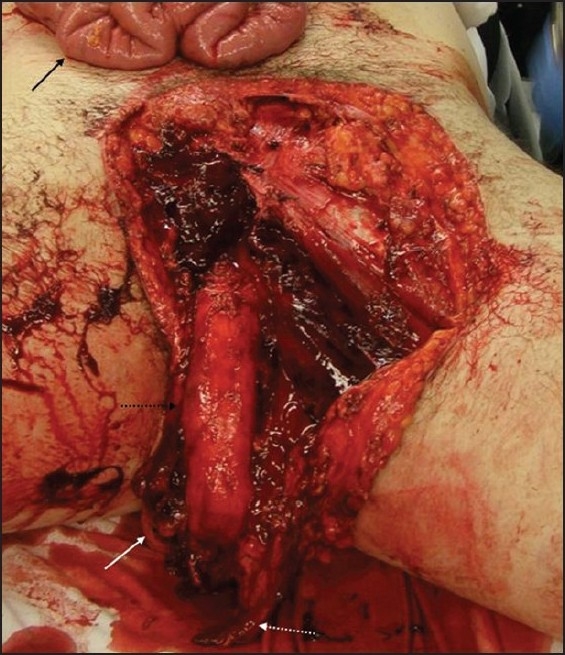
Injuries inflicted by a manure spreader. The groin laceration resulted in penis degloving (dashed black arrow), left lacerated spermatic cord (dashed white arrow), and right avulsed testicle (white arrow).The eviscerated bowel (black arrow) extrudes through an abdominal laceration (not shown)

The patient was met by our trauma team in the trauma bay and was found to have an eviscerated bowel wrapped in a moist towel extruding through a 12-cm curvilinear abdominal laceration 10 cm inferior to the costal margin extending vertically down the right midclavicular line. Additionally, the patient had an open pelvic fracture and a large curvilinear laceration extending from the right inferomedial portion of the inguinal area to the superior pubic symphysis and finally coursing to the left medial thigh resulting in degloving of the penis and bilateral testicular avulsion [Figure 1]. He was taken to the operating room immediately for exploration and wound care.

The trauma surgery team performed a trauma laparotomy and explored the abdominal cavity in addition to further assessment of the pelvic trauma. The urology team was able to concurrently examine the genital trauma. Both spermatic cords were completely severed. The team immediately performed testicular sperm extraction on the right testicle, collecting 21 vials of viable sperm, in an effort to retain the patient's future reproductive potential. It was determined that the left testicle was capable of revascularization.

As the trauma surgery team continued with the exploratory laparotomy, a discussion ensued regarding the priority and order in which the patient's surgery should be carried out. There was concern that revascularization of the testicle was time-sensitive and may be futile if delayed for an extended period of time. Ultimately, it was determined that the intra-abdominal portion would be completed, followed by open repair of the pelvic fracture by the orthopedic team and, finally, revascularization of the testicle by the plastic surgery team.

The laparotomy revealed the eviscerated small bowel through a large midline defect, a small bowel serosal and mesenteric tear, a circumferential sigmoid colon serosal tear and surprisingly sparse wound contamination, yielding only a few strands of vegetation. The small bowel mesenteric tear was oversewn but the sigmoid injury required partial colectomy and a primary anastomosis was performed.

**Figure 2:**
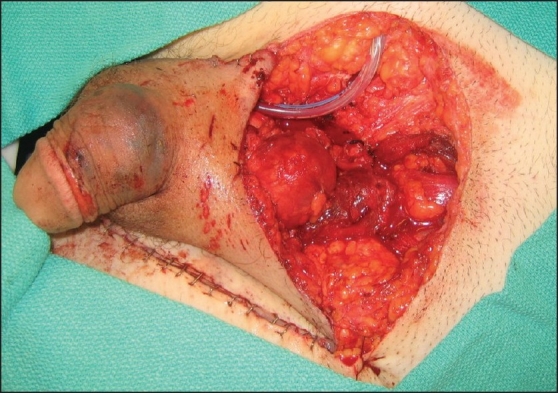
Partial repair of the inguinal injury including microsurgical anastomosis of left inferior epigastric artery and vein to the left testicular artery and vein respectively and testicular sperm extraction. The complex penile and scrotal lacerations were closed primarily

The urology team then performed a retrograde urethrogram, cystourethroscopy, cystogram and bilateral retropyelogram for evaluation of the urinary tract and placement of a Foley catheter. The urinary tract was found to be intact. Further, a pelvic X-ray revealed pubic symphysis diastasis, fracture of the left superior and inferior pubic rami and widening of the right sacroiliac joint. Orthopedics performed an open reduction and internal fixation of the pelvic fracture. Then, trauma surgery re-examined the abdominal cavity finding no evidence of hemorrhage or contamination. The midline fascial defect was re-approximated and the abdominal laceration and incision were closed. The complex penile and scrotal lacerations were closed primarily. Finally, plastic surgery successfully performed the revascularization of the left testicle through microsurgical anastomosis of the left inferior epigastric artery and vein to the left testicular artery and vein, respectively [Figure 2]. Arterial and venous Doppler flow with augmentation were present following the procedure and were marked for future detection.

The patient received eight units of blood, four units fresh frozen plasma and one unit platelets. The patient remained stable throughout the operation. Total time of operation was 8 h.

The patient was extubated on postoperative day 1 and discharged to rehabilitation 10 days after admission. The patient did not experience significant complications during his hospital course and was expected to make a near full recovery. Adequate blood flow was consistently demonstrated in the left testicle postoperatively by serial Doppler evaluation; however, testosterone levels were at castrate levels on day 1 and remained at this level until day 3, at which time the patient began applying daily topical testosterone gel.

Approximately 5 months post injury, the patient reported normal urinary, erectile and ejaculatory function. The patient continued to apply topical testosterone gel and declined any interest in determining the hormonal potential of his remaining testis. Testicular ultrasound (US) revealed a moderately atrophic left testicle with a necrotic-appearing center and absent central blood flow. Peripheral tissue appeared slightly heterogenic with normal arterial perfusion and venous return.

## DISCUSSION

This report highlights a case in which a patient was successfully surgically stabilized from a potentially life-threatening, polytraumatic injury, including significant genital trauma: penile degloving, bilateral testicular avulsion and bilateral spermatic cord laceration. Each genital injury described is rare by itself but together has not yet been documented. Therefore, each individual injury will be discussed separately and then, together, will be re-assessed, including their implications, with the attempt to restore reproductive and hormonal capabilities.

Penile degloving injuries commonly occur while using rotary-type machines typical in industrial and farm machinery. The rotary mechanism can catch loose clothing and in the process can catch and tear-off the redundant skin of the genitalia in what is referred to as a power take-off injury.[3,4] This injury has potential for significant complications, including infection, incontinence, erectile dysfunction and hypersensivity with subsequent loss of sensation, such that proper management is paramount.[5] Penile blood supply to superficial tissues is limited. Therefore, extragenital skin grafting is difficult to manage and increases the risk of complications, compared to primary closure, and should be limited to cases for which the native genital skin is irreparable.[6,7] The event skin grafting is needed. Thick, nonmeshed split-thickness skin grafts of the anterior thigh or buttocks are recommended.[8] In situations where native penile skin can be recovered, it is important to prevent desiccation by wrapping detached skin in moist towels.[9] McAninch *et al*. have shown that with early debridement, bowel and urinary diversion followed by proper penile and scrotal skin repair can result in acceptable cosmetic and functional results in all cases of major skin loss.[10] Our patient did not suffer from scrotal degloving. However, it should be noted that in the case of penile degloving, scrotal degloving often occurs and may have significant implications on gonadal function.[11] With the exception of functional impairment of the penis, i.e. erectile dysfunction and Peyronie's[7] disease, fertility and gonadal function should not be affected by penile degloving alone. Primary closure of our patient's penile degloving injury was completed with no evidence of complication and normal urinary, erectile and ejaculatory function on long-term follow-up.

Testicular avulsion is a general description of the tearing away of the testis from the scrotum. Occurrences are rare but do include animal bites,[5] gunshot wounds[12] and self-mutilation.[13] In our case, it appears that an impeller blade within the manure spreader impaled our patient and, in its designed motion, coursed through his pelvic region in a helical fashion, lacerating both spermatic cords and subsequently avulsing the testes from the scrotum. A similar case was reported in 1974 in which both testes were avulsed during a power take-off injury resulting in laceration of the bilateral spermatic cords. In this case, re-attachment was not attempted. In 1976, a microsurgical technique was described that allowed for undescended, intra-abdominal testis to be autotransplanted into the scrotum.[14,15] This technique paved the way for Lin *et al.* to document the first two known cases of successful replantation of traumatically avulsed testicles.[16] Since then, many other successful cases using this technique have been documented.[17–19] Interestingly, one case attempted a transplantation of an avulsed testis into the patient's bicep unsuccessfully.[20]

For functional and, if nothing else, psychological reasons, several authors have recommended an attempt to salvage testicular function versus orchiectomy.[8,16] The ischemic tolerance of the testis with respect to other parts of the genitalia is poor, which makes time to revascularization critically important. The lifespan of a devascularized testicle has been studied mostly in the setting of testicular torsion. It has been determined that after 24 h from the onset of symptoms, the likelihood of salvaging the hormonal and spermatogenic capabilities of the testicle is almost impossible. The ideal timeframe for restoring perfusion is within 4–6 h of symptom onset, with irreversible damage observed starting 1 h after onset. Maximum time of testis survival was observed 12 h after the onset of symptoms.[21] In our case, the restoration of the patient's right testis was deemed technically impossible; however, the revascularization of the left testicle was attempted. Perfusion to the testis was restored approximately 8 h after the time of the accident, with good Doppler flow present following the procedure and during initial follow-up visits. On postoperative day 3, the patient's testosterone was at castrate levels and subsequent exogenous testosterone treatment was initiated.

US, 5 months post injury, revealed a 10.4 mL left testis with a necrotic-appearing center but relatively normal-appearing tissue and blood flow observed peripherally. The significance of this imaging study is difficult to interpret. One study examining testicular torsion observed that individuals receiving orchiopexy versus orchiectomy had better testicular function, represented by increased inhibin B (reflects number of functioning Sertoli cells) and decreased follicle-stimulating hormone levels. Interestingly, the orchiopexy group, despite improved fertility markers, showed an average of 50% reduction of testicular volume of the affected testis at a 1-year follow up using US. Three cases of total testicular loss were reported in the orchiopexy group, which showed a 77–98% reduction at 1-year follow-up.[22] Normal testicular volume is 12–30 mL (average 18 mL) for an adult,[21] which, at 5 months, represents a 13–65% (average 42%) reduction in testicular volume in our patient. Further, exogenous testosterone is known to suppress the release of gonadotropic hormones and may result in significant testicular atrophy.[23] One study observed a 19.6% reduction in testicular volume in patients on exogenous testosterone therapy for only 6 months,[24] which may contribute to the atrophy likely observed in our patient. Taken together, it is possible that our patient has maintained some functionality of his testis given the following data. However, follow-up studies are needed to definitely determine this.

Preservation of fertility is a rapidly advancing branch of medicine, highlighting its great demand within the public and vital importance to individuals. Cryopreservation of sperm and testicular tissue has been successfully implemented in millions of patients suffering from conditions threatening their reproductive future.[25] The majority of these cases are for patients afflicted with cancer who are scheduled to undergo surgery that will threaten their reproductive capacity. A literature review did not reveal data discussing sperm or testicular tissue cryopreservation in patients suffering from genital trauma. One study examining the time interval of sperm harvestation in posthumous patients determined that collection of viable sperm may be considered up to 36 h after death.[26] Testicular sperm extraction in our patient produced 21 vials of viable sperm, with one to eight motile sperm per high-power field observed prior to cryopreservation. Although perfusion to the testes in a posthumous event does not instantaneously cease as is the case with spermatic cord laceration, the extended length of time for sperm survival represented by the posthumous study suggests that sperm harvestation 4 h after spermatic cord laceration (time elapsed between the accident and sperm harvest in our patient) should undoubtedly be considered. It should be noted that proper transportation is important to ensure the maximum success for this procedure. The testicle should be wrapped in moist gauze and placed in ice, without direct contact between the testicle and the ice.

Care for the polytraumatic patient can be complex and often requires the expertise of numerous medical specialties. It is has been shown that proper management of these patients, including prehospital care, rapid transport and life-saving surgical treatment, may improve the rate of preventable deaths from 20–30% to 2–9%.[2] Trauma algorithms serve as a guide to efficiently and effectively limit morbidity and mortality in the polytraumatic patient. Interventions to preserve the patient's vitals take first priority, followed by treatment for injuries that are not directly life-threatening but may become life-endangering or represent significant morbidity if not promptly addressed.[2,27] It is at this critical juncture that the primary provider needs to develop a plan and consult with specialists to effectively streamline the patient's care. The order of priority for patient care at this point is (1) brain injury, (2) eye and face injuries, (3) progressive spinal cord compression, (4) visceral organ injuries and (5) musculoskeletal injuries. In our case, the patient suffered life-threatening abdominal and pelvic injuries that required immediate attention. However, early in the management of these issues, both urology and plastics were consulted and provided potentially life-changing care to the patient that was not necessarily critical but addressed significant quality of life issues, including fertility and hormonal function.

## CONCLUSION

Although genitalia trauma is relatively rare, its management in polytrauma should not be overlooked. The number one priority in traumas will forever remain life-saving procedures. However, there is an increasing role in recognizing potential quality of life issues beyond the scope of a trauma surgery team. While heightened vigilance in recognition of fertility issues in penile degloving injuries can help spare reproductive and endocrine difficulties later in life, the recognition is useless without the active and immediate involvement of the appropriate surgical specialties. In our institution, we must appreciate the access to urology and plastics teams and utilize multidisciplinary care of our patient whenever appropriate.
